# The Action of Medicines in the System, or “On the Mode in Which Therapeutic Agents Introduced into the Stomach Produce Their Peculiar Effects on the Animal Economy”

**Published:** 1856-04

**Authors:** 


ART. III.— *The Action of Medicines in the System*, or "*On the Mode in which Therapeutic Agents introduced into the Stomach produce their peculiar Effects on the Animal Economy.*" Being the Prize Essay to which the Medical Society of London awarded the Fothergillian Gold Medal for 1852. By Frederick William Headland, M. B., B. A., F. L. S., M. R. C. S., etc. Second American from the Second Revised and Enlarged English Edition. Philadelphia: Lindsay & Blakiston. 1856.

The subject comprehended in this essay is so vast, and its bearings so extended, that the reviewer hesitates between a mere statement of its publication and a general endorsement of its value, and an extended analysis of the author's argument, in the confident expectation that the latter course, though lengthy, could not fail to benefit both his reader and himself.

He chooses, however, a different plan in confining himself to a mere statement of the theories propounded, with an occasional word of comment.

The author does not err in estimating highly the value of his subject. Diagnosis and nosology are rapidly attaining to the dignity of exact sciences. We know with what we deal in almost every case of disease which presents itself. We go over the chest of the invalid, and say to ourselves here is effusion, here is tubercle, here a cavity, here a pneumonia. We place the stethoscope upon his heart, and tell which of its different valves has failed in its office, or we map it out, and by a knowledge of its size may argue its abnormal weight. But when we give a remedy, we but cast a stone into Life's rapid current—the eddy may carry it far from its destination; we are fighting in the dark—striving against the elements.

Here—in the action of medicines—is a field for study, indeed. Here it is that we feel our weakness, for when we part with a medicine, as it passes down the open throat of a patient, we look upon it as we would upon a valued friend just starting for far-distant lands, perhaps to perish by the way, perhaps to come again with golden results.

This is the task of our author. He follows the drug into the unanswering walls of the inner man, and strives to trace its subtle chemistry there.

Dr. Headland's first proposition asserts that a remedy must, as a general rule, obtain entry into the fluids of the body before its action can begin. This he considers proven by the facts that medicines introduced through the skin act in the same manner as through the stomach; that poisons will not act through the medium of the nerves only, (as proven by experiment) but must have the intervention of the blood; that the circulation is so rapid as to allow ample time for the most rapid poison to perform the entire circuit before it begins to operate; and, finally, that medicines are detected by chemical analysis in the blood or secretions.

In the "Second Proposition," it is asserted that all those remedies which are soluble in water, pass through the coats of the stomach into the portal system of vessels, and that those which are, either wholly or in part, insoluble in water, as some of the minerals, resins, and fats, are dissolved by the alkaline secretions of the intestine. In most instances the act of solution effects but little change in the material absorbed. The albumens become simply albuminose, fats are only emulsionized, and not saponified; while in those numerous cases in which the pepsin or acids of the gastric juice form the active solvent, the change is usually one of degree only, and not of kind. All of this process of absorption is simply an endosmosis or exosmosis, the major current usually being from the alimentary tube to the vessels.

In a foot-note Dr. Headland states that "there is no doubt" that the capillary vessels of the intestines are capable of taking up any matters in a state of proper solution; but he opines that medicines are seldom or never taken up by the lacteals; "for it seems that these vessels are only engaged after a full meal and subsequent to the formation of chyle. They do not exist in the coats of the stomach," etc. It follows from this that all medicines pass through the portal system of veins, and, of course, the liver.

Those medicines which, by reason of insolubility, may not be absorbed, are discharged in the evacuations. To the discussion of this point the "Third Proposition" is devoted.

The "Fourth Proposition" states that a few substances may act locally on the mucous surface, by irritation or otherwise. This is stated to be a local effect only, to be perhaps succeeded by changes in distant parts, on the principle of revulsion.

How do medicines act *in* the blood? On this point the author says, on page 23 et seq:

"Having just shown how medicinal substances are absorbed, we have now to suppose they are in the blood.

"It is next maintained, in the *Fifth Proposition*, that the medicine, being in the blood, must permeate the mass of the circulation as far as to reach the part on which it tends to act. This it can easily do. The circulating blood will conduct it anywhere, in a very short time. Supposing a medicine has to act on the liver, or on the brain, or on the kidney, it does not influence these organs at a distance, but it passes directly to them in the blood, and then its operation is manifested. This may be called the rule of *local access.* Its proof depends on two things: on the improbability of the medicinal influence being able to reach the part in any other way, as shown in the first proposition; and on the fact of medicinal agents having been actually detected in many cases in the very organs over which they exert a special influence. But are there any exceptions to this? Can a medicine ever produce an effect without actually reaching the part? It seems that there may be two exceptions. In some cases an impression of *pain* may be transmitted along a nerve from one part to another; and in some other few instances a muscle, when caused to contract by the influence of a medicine, may cause other muscles near it to contract by sympathy.

"Before we inquire into the remedial action of the medicine in the blood, we must consider whether that fluid may not first alter it in some way, so as to hinder or affect its operation. To a certain extent this is possible.

"In the *Sixth Proposition* it is asserted, that while in the blood the medicine may undergo change, which change may or may not affect its influence

It will have to be shown that this change may be one of *combination*, as of an acid with an alkali; of *reconstruction*, when the elements of a body are arranged in a different way, without a material change in its medical properties, as when benzoic is changed into hippuric acid; or of *decomposition*, when a substance is altogether altered or destroyed, as when the vegetable acids are oxidized into carbonic acid.

"Having considered these preliminary matters, we shall arrive at the main point. The medicines are now in the blood. We must consider what becomes of them; what they do next; where they go next; and how they operate in the cure of diseases. I have made a classification in which medicines are divided according to my views of their mode of operation. The classes and their subdivisions will serve for references in illustration of what I have to say. For it is not possible to speak of the general operation of medicines without adducing partic lar instances; nor will time and space always allow me, in doing so, to refer to individual medicines.

"There are four great groups of medicines, the action of each of which is well marked and distinct. The first class acts in the blood; and as a large number of diseases depend on a fault in that fluid, we may by their means be enabled to remedy that fault. They are the most important of all medicines. They are called Hæmatics, or blood-medicines. They are used chiefly in chronic and constitutional disorders. But a second class of remedies are temporary in their action. They influence the nervous system, exciting it, depressing it, or otherwise altering its tone. They are chiefly useful in the temporary emergencies of acute disorders. They can seldom effect a permanent cure, unless when the contingency in which they are administered is also of a temporary nature. They are called Neurotics, or nerve-medicines. A third set of medicines, less extensive and less important than the others, acts upon muscular fibre, which is caused by them to contract. Involuntary muscular fibre exists in the coats of small blood-vessels, and in the ducts of glands. The Astringents, as these agents are called, are able, by contracting muscular fibre, and thus diminishing the calibre of these canals, to arrest hæmorrhage in one case (when a small vessel is ruptured,) and to prevent the outpouring of a secretion in another case.

"The fourth class is of considerable importance. Some medicines have the power of increasing the secretions which are formed from the blood by various glands at different parts of the body. By their aid we may be enabled to eliminate from the blood a morbid material through the glands; or we may do great good by restoring a secretion when unnaturally suppressed.

They are called Eliminatives. Like Hæmatics, their influence is more or less permanent. That of Neurotics and Astringents, particularly the former, is transient.

"The general mode of action of these four classes of therapeutic agents is laid down in the four remaining propositions, about as far as it seems to me to be capable of a positive definition. Each proposition concerns one of these classes of medicines. All I can do now is to recapitulate the chief affirmations made; as to give any idea of their proof would require me to enter into a number of details which had better be postponed to the third chapter.

In the *Seventh Proposition* it is stated of Hæmatic medicines that they act while in the blood, over which fluid they exert an influence; and that their effect, whatever it be, is of a more or less permanent character. A line of distinction is drawn between two divisions of this class of blood-medicines. Some of them are natural to the blood; they resemble or coincide with certain substances that exist in that fluid; so that, having entered it, they may remain there, and not necessarily be excreted again. These are useful when the blood is wanting in one or more of its natural constituents. This want causes a disease, and may be supplied by the medicine, which in this way tends to cure the disease. Medicines of this division are called Restoratives; for they *restore* what is wanting.

"Some other blood-medicines, although they enter the blood, are not natural constituents of the vital fluid, and cannot remain there, for they are noxious and foreign to it. They must sooner or later be excreted from it by the glands. They are of use when disease depends on the presence and working in the blood of some morbid material or agency, which material or action they tend to counteract or destroy. They may be called *vital antidotes;* not strictly *specifics*, for they are not always efficacious, on account of variations in the animal poisons, or from the casual operation of disturbing causes. They are applicable in those many disorders which depend, not on the absence of a natural substance, but on the presence of an unnatural agent in the blood. These medicines are called Catalytics, from a Greek word, which signifies t*o break up or destroy.* Having performed this their function, they then pass out of the blood.

"All this requires to be proved.

"In the *Eighth Proposition* it is stated of Neurotics, or nerve-medicines, that they act by passing out of the blood to the nerves, which they influence. This is only to insist on the rule of *local access*, already laid down in Prop. V. It is further affirmed that they are transitory in action. They appear to effect molecular changes in nerve-fibre, similar to those by which the phenomena of the senses are produced, and which are by nature transitory in their results. And yet they may be very powerful, even so as to extinguish vital force. Thus short and unenduring as is the operation of these agents, it may last long enough to cause death, and so a temporary influence produce a permanent result. There are three divisions of Neurotics. The first set are of use when there is a dangerous deficiency of vital action. These are Stimulants. They exalt nervous force, either of the whole nervous system, or only of a part of it. They vary very much in power. A second set, called Narcotics, first exalt nervous force, and then depress it. They have thus a double action; but they have also a peculiar influence over the functions of the brain, which is different from any possessed by other nerve-medicines. They control the intellectual part of the brain, as distinguished from its organic function; the powers of *mind* more than those of *life*. Some Narcotics tend to produce inebriation; others, sleep; others again, delirium. In the third place, some Neurotics tend simply and primarily to depress nervous force. They may act on the whole nervous system, or on a part of it only. They are often very powerful; and they are of use when, from any cause, some part of the nervous system is over-excited. They are called Sedatives. Like other Neurotics, they are used in medicine as temporary agents in temporary emergencies. If a permanent action be required, the remedy must be constantly administered, so that the effect may be kept up by continual repetition.

"In the *Ninth Proposition* it is affirmed of Astringent medicines that they act by passing out of the blood to muscular fibre, which by their contact they excite to contraction. They do not so much influence the voluntary fibre of the muscles, which is under the direct control of the nervous system; but they chiefly manifest their action on the involuntary or unstriped muscular fibre, which is not directly controled by the brain and nerve-centres, and for this reason more under the operation of external or irritating agents. Meeting this in the coats of the capillary vessels and of the ducts of glands, they are enabled to act as styptics, and as checkers of secretion. The action of Astringents appears to depend on a chemical cause; for we find that all of them possesses the power of coagulating albumen.

"The *Tenth Proposition* treats of Eliminatives. It is not said simply that these increase the secretions of a gland; or that they stimulate the glands while passing by them in the blood. But it is laid down as a rule that they act by themselves passing out of the blood through the glands, and that while so doing they excite them to the performance of their natural function·

They are substances which are unnatural to the blood, and must therefore pass out of it. In so doing they tend to pass by some glands rather than by others: in these secretions they may be detected chemically; and it is on these glands that they have an especial influence. Their uses in treatment are various and manifold.

"In these classes are included all medicines that act after entry into the blood. On referring to the classification which precedes this chapter, it will be seen at a glance what groups of medicines are arranged as orders under each class or division.* In the third chapter I shall attempt at some length to prove the propositions which treat of these four classes; and I shall also attempt to explain the nature and mode of action of the orders, or small groups of remedies.

"In the fourth chapter some of the more important medicines will be considered separately, either as individually interesting, or as illustrative of general modes of operation previously described.

"I may point to some parts of the Essay as being more original than the others, although not, perhaps, for that reason, more valuable. For this purpose may be mentioned the treatment of the second and third propositions; the distinction which I have drawn between the two divisions of Blood-medicines; the account given of Tonics in one of these divisions, and of Anti-arthritics in the other; the theory of the action of Eliminative medicines; the experiments made on the action of some remedies in particular (Chap. IV.), &c."

The liberal quotations we have given will convey to our readers the scope and tendency of Dr. Headland's work. In the discussion at length of the various propositions, he is logical and careful, and the reader cannot fail to acquire new ideas and facts which will possess a permanent value. It is, on the whole, one of the most interesting essays which have fallen under our notice.

---

* It may be of some use if I adduce here a characteristic example of each of the great groups of medicines to which I have alluded above:

Class I. *Hæmatics.*

- Div. 1. Restoratives. Iron, in Anæmia.

- Div. 2. Catalytics. Mercury, in Syphilis.

Class II. *Neurotics.*
- Div. 1. Stimulants. Ammonia.
- Div. 2. Narcotics. Opium.
- Div. 3. Sedatives. Hydrocyanic Acid.

Class III. *Astringents.* Tannic Acid.

Class IV. *Eliminatives.* Cantharides, and Croton Oil.

